# The potential role of nanobodies in asthma therapy

**DOI:** 10.3389/fphar.2024.1510806

**Published:** 2025-01-20

**Authors:** Baraa Khalid Salah Al-Sheakly, Fatemeh Saheb Sharif-Askari, Narjes Saheb Sharif-Askari, Jennifer E. Hundt, Rabih Halwani

**Affiliations:** ^1^ Research Institute for Medical and Health Sciences, University of Sharjah, Sharjah, United Arab Emirates; ^2^ Department of Pharmacy Practice and Pharmacotherapeutics, College of Pharmacy, University of Sharjah, Sharjah, United Arab Emirates; ^3^ Department of Clinical Sciences, College of Medicine, University of Sharjah, Sharjah, United Arab Emirates; ^4^ Lübeck Institute of Experimental Dermatology, University of Lübeck, Lübeck, Germany; ^5^ Department of Pediatrics, Faculty of Medicine, Prince Abdullah Ben Khaled Celiac Disease Chair, King Saud University, Riyadh, Saudi Arabia

**Keywords:** asthma, cytokine, inflammatory mediators, monoclonal antibodies, nanobodies (Nbs)

## Abstract

Asthma is a chronic inflammatory disease of the airways characterized by bronchoconstriction, airway hyperresponsiveness, and mucus production. The pathophysiology of asthma involves a complex interplay of immune cells and mediators, including cytokines, chemokines, and other inflammatory molecules. Despite advances in asthma management, many patients continue to experience symptoms due to the limitations of current therapies. Monoclonal antibodies (mAbs) targeting specific inflammatory mediators have improved treatment outcomes for some patients, but challenges such as poor tissue penetration and high costs remain. Nanobodies (Nbs), a novel class of single-domain antibodies, offer a promising alternative due to their small size, stability, and potential for enhanced tissue penetration. This review discusses the key mediators involved in asthma, challenges in current treatments, and the potential of Nbs as a new therapeutic strategy. We also explore current studies and innovations in nanobody technology.

## 1 Introduction

Asthma is a heterogeneous disease characterized by chronic inflammation of the airways, leading to symptoms such as wheezing, shortness of breath, chest tightness, and coughing (Mims, 2015). The inflammatory response in asthma is driven by various immune cells and mediators, particularly in response to allergens and other triggers. Standard treatments for asthma focus on controlling inflammation and relieving bronchoconstriction (Mims, 2015). Inhaled corticosteroids (ICS) are the cornerstone of anti-inflammatory therapy, often combined with long-acting beta-agonists (LABAs) to manage chronic symptoms (Tashkin et al., 2021). For patients with severe asthma (Israel and Reddel, 2017), who do not respond adequately to these therapies (Dreher and Muller, 2018), biologic treatments have been developed. Monoclonal antibodies (mAbs) targeting specific cytokines, such as IL-5, IL-4/IL-13, and IgE, have provided significant benefits for patients with specific asthma phenotypes (Kardas et al., 2022). For example, mAbs like mepolizumab (anti-IL-5), dupilumab (anti-IL-4/IL-13), and omalizumab (anti-IgE) have improved outcomes in patients with eosinophilic and allergic asthma, respectively (Koski and Grzegorczyk, 2020). Despite the effectiveness of mAbs in treating asthma, their use is associated with several challenges (Abe et al., 2021) that nanobodies could potentially overcome (Paul et al., 2023). In this review, we will discuss the key inflammatory mediators involved in asthma, the current treatments for asthma and the associated challenges in managing the disease, and how nanobodies offer a promising new avenue for therapy. We will explore the latest research on nanobody development, including how these novel molecules can target the same mediators as existing mAbs but with potentially improved outcomes. The review will also highlight the ongoing clinical studies and innovations in nanobody technology that may shape the future of asthma treatment.

## 2 Key inflammatory mediators in asthma

Asthma pathophysiology involves a complex interplay of cytokines and chemokines that orchestrate the immune response, leading to chronic inflammation, airway hyperresponsiveness, and remodelling. These mediators are produced by various immune cells, including T-helper cells, eosinophils, mast cells, and macrophages, and play distinct roles in the development and exacerbation of asthma symptoms (Sinyor and Concepcion Perez, 2024; Peebles and Aronica, 2019).

Key cytokines include interleukins (ILs), tumour necrosis factor-alpha (TNF-α), and interferons (IFNs). IL-1β and IL-6 are crucial in initiating and propagating inflammatory responses by promoting the recruitment of immune cells to lung tissue (Lambrecht et al., 2019). TNF-α increases the permeability of endothelial cells, aiding the extravasation of immune cells into inflamed lung tissue (Al-Qahtani et al., 2024). Interferons, particularly IFN-γ, are essential for activating macrophages and promoting the adaptive immune response (Rich et al., 2020).

Chemokines, a subset of cytokines, specifically direct the migration of immune cells to inflammation sites. Chemokines such as CXCL8 (IL-8), CCL2 (MCP-1), and CCL5 (RANTES) are significantly upregulated during asthma-related lung inflammation. CXCL8 is a potent chemoattractant for neutrophils, while CCL2 recruits monocytes, and CCL5 attracts T cells, eosinophils, and basophils to the inflammation site (Lukacs, 2001; Lukacs et al., 1999). These molecules not only help in recruiting immune cells but also activate them, enhancing their ability to combat inflammation and contributing to asthma symptoms (Lukacs et al., 1999).

Balancing and regulating these cytokines and chemokines is crucial, as their dysregulation can lead to chronic inflammation and tissue damage, contributing to the persistence and severity of asthma (Lukacs et al., 1999). Elevated levels of IL-13, IL-4, and IL-5 are particularly associated with asthma, contributing to airway hyperresponsiveness, eosinophil recruitment, and mucus production (Pelaia et al., 2022; Nakagome and Nagata, 2024). IL-33 and thymic stromal lymphopoietin (TSLP) further amplify these responses by promoting the release of type 2 cytokines and enhancing IgE production, a hallmark of allergic asthma (Calderon et al., 2023; Stanbery et al., 2022). IL-17, produced by Th17 cells, plays a dual role in asthma (Wang and Wills-Karp, 2011). While it helps recruit neutrophils to combat extracellular pathogens, its overproduction can exacerbate airway inflammation and contribute to the severity of asthma (Rahmawati et al., 2021).

## 3 Current treatments for asthma

Current treatments for asthma focus on reducing airway inflammation and preventing exacerbations. Inhaled corticosteroids (ICS), such as fluticasone and mometasone furoate, are foundational therapies that diminish inflammation by inhibiting cytokine production and the recruitment of immune cells (Barnes, 2010). Combination inhalers that pair ICS with long-acting beta-agonists (LABAs), like fluticasone/salmeterol (Zhang et al., 2022) and budesonide/formoterol (Kew et al., 2013), provide enhanced benefits by concurrently reducing inflammation and inducing bronchodilation (Zhang et al., 2022; Kew et al., 2013). For patients with severe asthma unresponsive to standard therapies, monoclonal antibodies (mAbs) offer targeted intervention (Lyly et al., 2020). Biologics such as omalizumab (anti-IgE) (Kotoulas et al., 2022), mepolizumab (anti-IL-5) (Farne et al., 2017), and dupilumab (anti-IL-4R) (Harb and Chatila, 2020) have demonstrated efficacy in decreasing exacerbation rates and improving lung function by specifically modulating key inflammatory pathways involved in asthma pathogenesis (Kotoulas et al., 2022; Farne et al., 2017; Harb and Chatila, 2020).

## 4 Challenges in the treatment of asthma

Despite the availability of targeted therapies, several challenges persist in asthma treatment (Caminati et al., 2021). One of the major hurdles is achieving effective pulmonary drug delivery (Labiris and Dolovich, 2003a). This involves not only ensuring that medications reach the specific target sites within the lungs but also minimizing systemic exposure and potential side effects (Labiris and Dolovich, 2003a). Inhalation is the preferred route for delivering asthma medications, providing direct access to the respiratory tract and a rapid onset of action. However, barriers such as mucus, mucociliary clearance, and the alveolar-capillary barrier can hinder drug deposition in the lungs (Guo et al., 2021; Labiris and Dolovich, 2003b).

Particle size is a critical factor for effective drug delivery. Aerosolized particles that are too large may deposit in the oropharynx and be swallowed, whereas particles that are too small might be exhaled before reaching deep lung regions (Thomas, 2013). Optimal particle size for deep lung deposition is typically between 1 and 5 μm (Labiris and Dolovich, 2003b). The heterogeneous structure of the lungs, with its branching airways and varying airflow dynamics, further complicates uniform drug distribution (Fei et al., 2023). Techniques such as using propellants in metered-dose inhalers (Holland et al., 2013) or designing dry powder inhalers and nebulizers are employed to enhance delivery efficiency, but each method has its limitations (Ye et al., 2022).

Pharmacokinetics also significantly impacts the effectiveness of asthma drug delivery (Derendorf et al., 2006). Medications must be efficiently absorbed across the respiratory epithelium to achieve therapeutic levels (Labiris and Dolovich, 2003a). Factors such as the presence of lung surfactants, enzymatic degradation, and rapid clearance through the lymphatic system or bloodstream can reduce drug bioavailability (Labiris and Dolovich, 2003a). Additionally, patient-related factors including inhalation technique, lung capacity, and adherence to therapy influence treatment outcomes (Ma et al., 2023).

These challenges underscore the need for innovative strategies to improve lung-targeted drug delivery. Approaches such as developing nanoparticles and liposomes for better drug encapsulation, protection against enzymatic degradation, and sustained release, as well as designing personalized inhaler devices, are being explored to enhance therapeutic efficacy and minimize systemic side effects (Cheng et al., 2023; Liu et al., 2022). In this context, nanobodies—small single-domain antibody fragments derived from camelid antibodies—emerge as a promising solution (Arbabi-Ghahroudi, 2022).

## 5 Nanobodies: history, structure and characteristics

### 5.1 History of nanobodies

**FIGURE 1:**
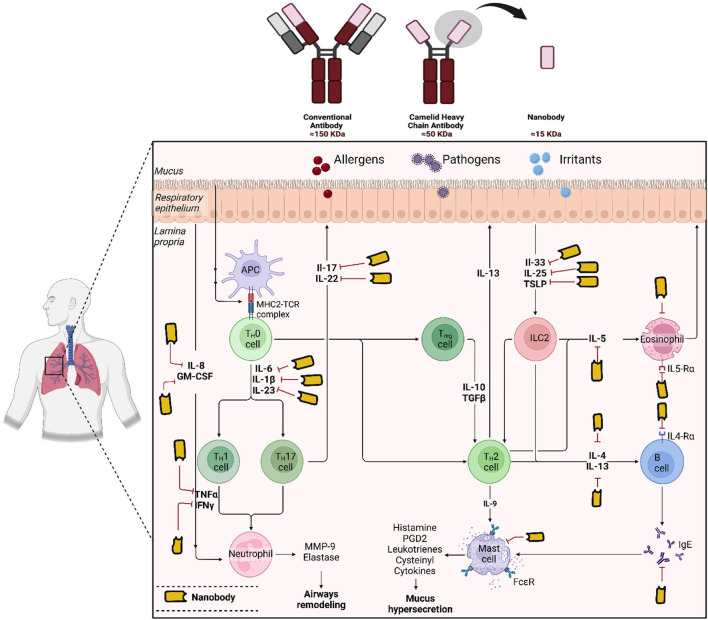
Schematic representations illustrate the structures of conventional antibodies (IgG), heavy-chain-only antibodies, and nanobodies (VHH). The potential targets of nanobodies in asthma treatment, are also indicated. The figure was generated using BioRender.com.

Antibodies are traditionally defined as molecules with two heavy chains and two light chains. However, there was an important change in the traditional understanding of antibodies in 1989. This research conducted by Professor Raymond Hamers of the Vrije University Brussel (VUB) resulted in the unexpected discovery of heavy chain-only antibodies (HCAbs) which lack a light chain (Figure 1). This discovery happened *via* student-led research which formulated a sero-diagnostic assay in order to diagnose trypanosome infection in camels and water buffalos (Muyldermans, 2013).

The discovery of Camelid heavy-chain antibodies has prompted widespread interest in utilizing these antibody domains in a variety of applications such as research, diagnostics and therapeutics (Muyldermans, 2013). These camelid heavy-chain antibodies are also known as VHHs/sdAbs/nanobodies (Figure 1). The formation of camelid VHHs for medicinal purposes occurred in three separate stages. The first decade (1993–2003) could potentially be considered as the exploration period (Arbabi-Ghahroudi, 2017). During 1996 and 2001, numerous patents were granted to research institutions in Belgium and Netherlands with an emphasis on potential commercial uses. Ablynx was established in 2001 with the primary goal of advancing nanobody-based medications and examining their therapeutic potential (Arbabi-Ghahroudi, 2017).

During the period of 2003–2013, a significant increase in publications surpassing 1,000 by 2013 was observed which suggests a substantial increase in attention and research focus on VHHs (Arbabi-Ghahroudi, 2017). There has been an evident increase in publications throughout the current developmental period starting from 2014 to the present and numerous VHHs have progressed into clinical trials or are getting ready for market release (Arbabi-Ghahroudi, 2017). Two decades of continuous work by Ablynx led to the formulation of the first nanobody medication known as caplacizumab (Cablivi) (Scully et al., 2019). The approval was obtained from the European Medicines Agency (EMA) and the Food and Drug Administration (FDA) in 2018 and 2019, respectively (Bergstrand et al., 2022). This novel drug cures a rare blood clotting disorder called the acquired thrombotic thrombocytopenic purpura (TPP) (Scully et al., 2019). Multiple variables are accountable for the long duration that passed between the discovery of camelid single-domain antibodies (sdAbs) and their release into the market. One of the major variables is the novel nature of this approach**.**

### 5.2 Structure and characteristics of nanobodies

Camelidae species are immunized against specific targets or antigens which result in the production of heavy chain antibodies (HCAb) and conventional antibody repertoires *in vivo*. Phage-display libraries provide a reliable representation of the various in vivo-matured heavy chain repertoires since they are generated by cloning amplified VHH repertoires with barely any alteration (Arbabi-Ghahroudi, 2017).

The remarkable specificity and affinity of VHHs are similar to those of conventional antibodies. Also, they exhibit excellent solubility, stability at different temperatures and possess monomeric behavior (Ikeuchi et al., 2021). VHHs are extremely tiny, measuring around 2.5 nm in diameter and 4 nm in length with a molecular weight of about 15 kDa (Hoey et al., 2019). They are easier to genetically engineer and can easily be produced for a relatively low price (Hoey et al., 2019). Moreover, they exhibit low immunogenicity and have improved tissue penetration properties (Khodabakhsh et al., 2018).

The remarkable thermostability of nanobodies is demonstrated by their capacity to retain 80% of their activity even after exposure to 37°C for a week (Paul et al., 2023). Furthermore, they exhibit resistance to proteases, denaturing agents and high pH levels (Paul et al., 2023). Despite their extremely short development time, research suggests that nanobodies can be generated in large quantities employing a microbiological system (de Marco, 2020). Nanobodies offer a promising alternative to conventional antibodies in disease diagnosis and treatment due to their unique advantages.

## 6 Nanobodies in asthma treatment

Ongoing *in silico*, preclinical studies, and clinical trials are advancing the role of nanobodies in asthma treatment, presenting promising alternatives to traditional monoclonal antibodies (mAbs) as summarized in Table 1.

**TABLE 1:** Summary of key studies on nanobody development in asthma treatment.

Study type	Objectives	Key findings	Conclusion	References
In Silico Development	To design and optimize nanobody-based therapeutics for asthma using computational tools	Utilized molecular dynamics simulations and homology modeling to improve stability, solubility, and specificity of nanobodies. Engineered single-domain antibodies showed improved binding affinity, stability, and solubility	In silico methods can effectively design stable, high affinity nanobodies for asthma treatment	Araújo et al. (2023)
Preclinical Development	To develop a bispecific nanobody targeting IL-5 and albumin for enhanced efficacy in asthma treatment	The bispecific nanobody showed 58 times higher efficacy than current IL-5 therapies, with excellent pharmacokinetics and sustained eosinophil suppression	The bispecific nanobody could be a next-generation therapy for eosinophilic asthma	Ma et al. (2022)
Preclinical Development	To engineer inhalable nanobodies targeting IL-5 for asthma treatment	AIL-A96-Fc effectively blocked the IL-5/IL-5Rα interaction and demonstrated cross-species activity with human and cynomolgus IL-5	AIL-A96-Fc shows promise as an inhaled therapeutic for eosinophilic asthma	Shijie et al. (2024)
Preclinical Development	To produce a bispecific nanobody targeting both IL-4Rα and IL-5	The bispecific nanobody inhibited IL-4, IL-5, and IL-13 interactions, showing enhanced therapeutic potential compared to dupilumab	Bispecific antibodies could improve efficacy in treating asthma by targeting multiple cytokines	Qiu et al. (2024)
Preclinical Development	To design an inhalable nanobody targeting IL-4Rα for asthma treatment	LQ036 effectively inhibited asthma-related biomarkers, reduced airway inflammation, and showed favorable pharmacokinetics and safety	LQ036 could be an effective inhalable biologic for asthma treatment	Zhu et al. (2024)
Preclinical Development	To develop nanobodies targeting IL-13 for better asthma management	Multimeric nanobodies showed enhanced affinity and biological activity, improving IL-13 inhibition	Multimeric nanobodies offer a more effective approach for targeting IL-13 in asthma	Gevenois et al. (2021)
Preclinical Development	To develop a bispecific nanobody targeting IgE and human serum albumin for asthma treatment	ALX-0962 effectively neutralized IgE and displaced preformed IgE-FcεRI complexes, reducing basophil degranulation	ALX-0962 may provide faster clinical improvement in asthma with dual functionality	Rinaldi et al. (2013)
Preclinical Development	To develop Bet v 1-specific nanobody trimers for preventing allergic reactions	Nanobody trimers showed enhanced cross-reactivity and better inhibition of IgE-allergen interactions than monomers	Nanobody trimers could be a promising strategy for preventing allergic reactions	Bauernfeind et al. (2024)
Preclinical Development	To produce an anti-IgE nanobody from the Indian dromedarius camel for asthma	The nanobody significantly reduced IgE production and alleviated airway inflammation, bronchoconstriction, and hyperresponsiveness in a mouse model	This camelid-derived nanobody may be an effective therapeutic strategy for allergic inflammation	Paul et al. (2023)
Clinical Trial	To evaluate the safety and efficacy of SAR443765, a bifunctional nanobody targeting TSLP and IL-13	A single dose of SAR443765 significantly reduced FeNO, IL-5, and IgE levels, with improvements in FEV1. The treatment was well-tolerated	SAR443765 shows potential as a groundbreaking therapeutic for type 2 asthma	Deiteren et al. (2023)

### 6.1 In silico nanobodies development in asthma

Recent advances *in silico* approaches have greatly contributed to the design and optimization of nanobody based therapeutics for asthma. Using computational tools such as molecular dynamics simulations and homology modeling (Cheng et al., 2019), researchers have focused on designing single-domain antibodies with enhanced stability, solubility, and specificity (Cheng et al., 2019).

One study utilized a camelization approach to create three specific mutated single-domain antibodies targeting a key pro-inflammatory cytokine implicated in allergic asthma. Using a monoclonal antibody structure as a template, these mutations significantly improved solubility and stability. Simulations revealed stable, long-lasting interactions mediated primarily by complementary-determining regions (CDRs). The engineered single-domain antibodies demonstrated improved binding affinity, stability, and solubility compared to their wild-type counterparts, highlighting their therapeutic potential (Araújo et al., 2023).

### 6.2 Preclinical nanobodies development in asthma

In recent preclinical studies, several promising nanobody-based therapies have been developed for the treatment of asthma and related allergic conditions, focusing on different therapeutic targets. For instance, Ma, L. et al. developed a trivalent bispecific nanobody targeting IL-5 and albumin to improve efficacy and address limitations of current IL-5 therapies (Ma et al., 2022). This nanobody showed superior efficacy over existing IL-5 therapies like mepolizumab, being 58 times more effective in inhibiting TF-1 cell proliferation. It also demonstrated excellent pharmacokinetics and sustained eosinophil suppression in primates. These results suggest the nanobody’s potential as a next-generation therapeutic for severe eosinophilic asthma, offering improved efficacy and longer-lasting effects (Ma et al., 2022). Similarly, Li, Shijie et al. engineered nanobodies suitable for inhalation administration that target IL-5, a cytokine critical for eosinophil proliferation and activation. Among the candidates, AIL-A96-Fc was identified as a highly effective nanobody that blocked the IL-5/IL-5Rα interaction and demonstrated cross-species activity with both human and cynomolgus IL-5. AIL-A96-Fc exhibited significant blocking effects, underscoring its potential as an inhaled therapeutic for eosinophilic asthma (Shijie et al., 2024).

Additionally, Qiu, W. et al. produced a bispecific antibody targeting both IL-4Rα and IL-5, utilizing humanized VHHs derived from alpacas (Qiu et al., 2020). They further investigated the epitope interactions of these VHHs with IL-4Rα and IL-5. Structural and biochemical analyses demonstrated that the nanobodies effectively inhibited the interactions between IL-4, IL-5, IL-13, and their respective receptors. Compared to dupilumab, which targets only IL-4Rα and has limited efficacy in severe disease, this bispecific antibody simultaneously attenuates the activity of three cytokines (IL-4, IL-5, and IL-13), offering enhanced therapeutic potential (Qiu et al., 2020).

Furthermore, Zhu, M. et al. designed an inhalable nanobody (Nb) targeting the IL-4Rα chain for asthma treatment, capitalizing on the inherent stability and efficacy advantages of nanobodies. By utilizing three immunized Nb libraries, they created the bivalent Nb, LQ036, which exhibited high affinity and specificity for human IL-4Rα. Preclinical tests in humanized mice demonstrated that LQ036 effectively inhibited key asthma-related biomarkers, including IgE and CCL17, reduced airway inflammation, and showed favourable pharmacokinetics and safety profiles. These findings underscore the potential of LQ036 as an effective inhalable biologic for the treatment of asthma (Zhu et al., 2024).

Meanwhile, Gevenois, P. J. Y. et al. developed nanobodies targeting IL-13, a key cytokine in allergy, inflammation, and fibrosis. While the initial nanobodies showed good affinity, they were ineffective at inhibiting IL-13 biological activity *in vitro*. To enhance efficacy, multimeric constructs were created, resulting in a significant increase in both affinity and biological activity, suggesting that multimeric nanobodies could be a promising approach for more effective IL-13 targeting (Gevenois et al., 2021).

In a similar manner, Rinaldi, M. et al. constructed ALX-0962, a bispecific nanobody targeting IgE and human serum albumin to extend plasma half-life (Rinaldi et al., 2013). Unlike Omalizumab, ALX-0962 demonstrated dual functionality, effectively neutralizing soluble IgE with higher potency while displacing preformed IgE-FcεRI complexes on basophils. This dual action significantly reduced basophil degranulation at nanomolar concentrations. These findings highlight ALX-0962s potential to provide a faster onset of clinical improvement in asthma treatment (Rinaldi et al., 2013).

In addition, Bauernfeind, C. et al. developed high-affinity Bet v 1-specific nanobody trimers to outcompete IgE binding and prevent allergic reactions. The engineered trimers showed enhanced cross-reactivity, slower dissociation rates, and better inhibition of IgE-allergen interactions compared to monomers. They effectively reduced IgE binding to Bet v 1 and related allergens while suppressing allergen-induced basophil degranulation. These results highlight the potential of nanobody trimers as a promising therapeutic strategy to prevent allergic reactions caused by Bet v 1 and its cross-reactive allergens (Bauernfeind et al., 2024).

Likewise, a study produced an anti-IgE nanobody derived from the Indian dromedarius camel to reduce hypersensitivity in allergic asthma. Using an ovalbumin-induced mouse model, the nanobody significantly suppressed IgE production and alleviated symptoms of airway inflammation, including bronchoconstriction and airway hyperresponsiveness. The results suggest that this camelid-derived nanobody could be a promising therapeutic strategy for allergic inflammation (Paul et al., 2023).

### 6.3 First clinical study of nanobodies in asthma

SAR443765, developed by Sanofi, is the first and only nanobody to date to reach a Phase 1 clinical trial for asthma treatment, marking a significant advancement in biologics targeting type 2 airway inflammation (Deiteren et al., 2023). This bifunctional NANOBODY^®^, designed to block both TSLP and IL-13, demonstrated promising safety and efficacy results in the trial (NCT05366764). In 36 mild-to-moderate asthma patients with elevated FeNO, a single subcutaneous dose significantly reduced FeNO at week 4, outperforming the effects of monovalent biologics targeting either pathway. Reductions in blood biomarkers, such as IL-5 and IgE, aligned with these findings, and numerical improvements in prebronchodilator FEV1 were observed. The treatment was well-tolerated, with only mild to moderate Treatment-emerging adverse events such as nasopharyngitis and injection site reactions. These results highlight SAR443765s potential as a groundbreaking therapeutic for asthma (Deiteren et al., 2023).

The advancement of SAR443765 into clinical trials marks a significant milestone, demonstrating the transformative potential of nanobodies as promising therapeutic agents for asthma. This success highlights the urgent need for further research and development to translate more preclinical breakthroughs into clinical applications, paving the way for nanobodies to revolutionize asthma treatment and address critical unmet medical needs.

#### 6.3.1 Nanobodies in various diseases and their potential use for asthma treatment

Nanobodies are demonstrating considerable potential across a spectrum of diseases, for instance, M1095, an anti-IL-17A/F nanobody, has shown effectiveness in treating moderate-to-severe plaque psoriasis by targeting IL-17A and IL-17F, which are also involved in severe asthma (Svecova et al., 2019). Furthermore, ALX-0061, a bispecific nanobody that targets the IL-6 receptor (IL-6R), is used for conditions involving excessive IL-6 signalling, such as rheumatoid arthritis (Van Roy et al., 2015). Similarly, Sonelokimab, which targets both IL-17A and IL-17F, shows promise in treating Hidradenitis Suppurativa (Hunt et al., 2023). ALX-0171, a 42 kDa trivalent nanobody currently used in nebulizer solutions for respiratory syncytial virus (RSV) infections, targets the fusion (F) protein of RSV with high affinity, effectively inhibiting viral replication (Detalle et al., 2016).

M1095, Sonelokimab, ALX-0061, and ALX-0171, though initially developed for conditions like psoriasis, Hidradenitis Suppurativa, rheumatoid arthritis, and RSV infections respectively, exhibit considerable potential for asthma treatment. M1095 could be repurposed to target IL-17A and IL-17F in asthma, potentially reducing inflammation (Wang and Wills-Karp, 2011). ALX-0061, with its ability to neutralize IL-6R, might be adapted to address IL-6 in asthma (Rincon and Irvin, 2012). Similarly, ALX-0171s mechanism for RSV could provide insights into managing asthma exacerbations related to viral infections (Rosas-Salazar et al., 2023). These nanobodies, originally designed for other diseases, demonstrate versatile mechanisms that make them promising candidates for innovative asthma therapies.

#### 6.3.2 Future directions for nanobodies in asthma treatment

The future of nanobodies in asthma treatment is set to bring innovative solutions, addressing both clinical and therapeutic gaps in current asthma management.

• Expansion of Targeted Inflammatory Mediators: Currently, nanobody based therapies primarily target mediators such as IL-4, IL-5 and IgE. However, the expansion of this therapeutic approach to include other inflammatory biomarkers such as IL-1β, IL-6, IL-25, IL-33, and TGF-β presents an opportunity to manage more severe and resistant forms of asthma, including steroid hyporesponsive asthma (Lambrecht et al., 2019; Calderon et al., 2023; Stanbery et al., 2022; Sim et al., 2024). These molecules are involved in various stages of the inflammatory response in asthma and could offer more comprehensive control over the disease’s complex pathophysiology (Mims, 2015). By targeting multiple cytokines, nanobodies could prevent the exacerbation of asthma symptoms in patients who do not respond well to current treatments.
• Combination Therapies: The use of nanobodies in combination with existing therapies, such as corticosteroids, biologics, or bronchodilators, could enhance treatment efficacy (Jovcevska and Muyldermans, 2020). Nanobodies may address multiple inflammatory pathways simultaneously, increasing the effectiveness of asthma treatment (Jovcevska and Muyldermans, 2020). Combination therapies could help tackle both the underlying inflammatory mechanisms and the symptoms of asthma, offering a more holistic approach to management (Saleh, 2008).
• Targeted Delivery Systems: Aerosolized nanobodies, designed for direct pulmonary delivery, are an exciting direction for the future of asthma treatment (Van Heeke et al., 2017; Mustafa and Ahmed, 2023). This delivery method ensures that nanobodies are precisely targeted to the lungs, enhancing therapeutic efficacy while minimizing systemic side effects (Labiris and Dolovich, 2003a). Aerosolized nanobodies could improve treatment compliance by offering a more convenient and localized approach to asthma management (Labiris and Dolovich, 2003a).
• Improving Stability and Delivery Mechanisms: Nanobody stability and pharmacokinetics are critical factors for their clinical application. Current research is focused on improving the shelf-life, stability, and delivery of nanobodies through advanced formulations (Mir et al., 2020; Dingus et al., 2022). These innovations may include using engineered carriers or nanoparticles to enhance the bioavailability and efficacy of nanobodies, allowing for sustained release and optimal dosing intervals (Dingus et al., 2022). Such advancements would make nanobody treatments more effective and easier to administer, contributing to better patient outcomes (Dingus et al., 2022; Jin et al., 2023; Kunz et al., 2018).
• Long-Term Studies and Clinical Evaluation: While preclinical studies have shown promising results, long-term clinical studies are necessary to fully assess the safety, efficacy, and potential side effects of nanobody based asthma treatments (Jovcevska and Muyldermans, 2020). These studies should focus on evaluating sustained benefits and how nanobodies perform over extended periods of use. Furthermore, clinical trials should explore their impact on lung function, symptom control, and quality of life in patients with asthma. Only through comprehensive clinical evaluation can the full potential of nanobodies be realized.
• Cost-Effectiveness and Accessibility: As with any novel therapeutic, the cost of nanobody based treatments must be considered. Research is underway to identify ways to make nanobodies more cost-effective, which would increase accessibility to a larger number of patients (Fridy et al., 2014). Reducing the cost of nanobodies could make them viable alternatives to current expensive biologic therapies, providing patients with more affordable options for managing asthma (Fridy et al., 2014). Ensuring these treatments are widely accessible will be key to their adoption and success in clinical practice.

In summary, the future of nanobodies in asthma treatment holds immense promise. From targeting multiple inflammatory mediators and advancing personalized medicine to improving delivery systems and reducing treatment costs, these developments will shape the next-generation of asthma therapies. Continued research and clinical trials are essential to fully realize the potential of nanobodies and improve outcomes for asthma patients worldwide.
